# Intraspecific lineage divergence and its association with reproductive trait change during species range expansion in central Eurasian wild wheat *Aegilops tauschii* Coss. (Poaceae)

**DOI:** 10.1186/s12862-015-0496-9

**Published:** 2015-09-30

**Authors:** Yoshihiro Matsuoka, Shigeo Takumi, Taihachi Kawahara

**Affiliations:** Fukui Prefectural University, Matsuoka, Eiheiji, Yoshida, Fukui Japan; Laboratory of Plant Genetics, Graduate School of Agricultural Science, Kobe University, Nada-ku, Kobe, Japan; Laboratory of Crop Evolution, Plant Germ-plasm Institute, Graduate School of Agriculture, Kyoto University, Mozume, Muko, Kyoto Japan

**Keywords:** Adaptation, Dispersal, Flowering time, Life history, Natural variation, Phenology, Spike and spikelet traits, Species range expansion

## Abstract

**Background:**

How species ranges form in landscapes is a matter of long-standing evolutionary interest. However, little is known about how natural phenotypic variations of ecologically important traits contribute to species range expansion. In this study, we examined the phylogeographic patterns of phenotypic changes in life history (seed production) and phenological (flowering time) traits during the range expansion of *Aegilops tauschii* Coss. from the Transcaucasus and Middle East to central Asia.

**Results:**

Our comparative analyses of the patterns of natural variations for those traits and their association with the intraspecific lineage structure showed that (1) the eastward expansion to Asia was driven by an intraspecific sublineage (named TauL1b), (2) high seed production ability likely had an important role at the initial dispersal stage of TauL1b’s expansion to Asia, and (3) the phenological change to early flowering phenotypes was one of the key adaptation events for TauL1b to further expand its range in Asia.

**Conclusions:**

This study provides for the first time a broad picture of the process of *Ae. tauschii*’s eastward range expansion in which life history and phenological traits may have had respective roles in its dispersal and adaptation in Asia. The clear association of seed production and flowering time patterns with the intraspecific lineage divergence found in this study invites further genetic research to bring the mechanistic understanding of the changes in these key functional traits during range expansion within reach.

**Electronic supplementary material:**

The online version of this article (doi:10.1186/s12862-015-0496-9) contains supplementary material, which is available to authorized users.

## Background

The borders of species’ geographic ranges may shift over time due to habitat expansion, contraction, fragmentation, and merger. Species ranges expand through the migration of individuals from their native habitats to new territories, whereas local population extinction may cause range size reduction. How species ranges form in landscapes is a matter of long-standing evolutionary interest [1–3]. From a broad ecological viewpoint, there are two important drivers for species range expansion: dispersal and adaptation. In fact, range expansion ceases when dispersal or adaptation or both are limited in the range-edge populations [4, 5]. Various forms of dispersal constraints, including temporal and spatial barriers, low innate dispersal ability, and absence of dispersal vectors, reduce the chance for a species to migrate to novel suitable habitats outside the range even if they exist. The causes for limited adaptation that makes species less fit in the environmental conditions of newly introduced territories can be genetic (e.g., inadequate levels of genetic variation for target traits in response to the act of natural selection) or ecological (e.g., trade-offs that involve the traits of ecological importance). Ecological studies have provided empirical evidence for negative impacts of limited dispersal and adaptation on range expansion in various plant and animal species [6–10]. In contrast, many genetic studies on the mechanisms that underlie the adaptive change in ecologically important traits (including dispersal traits) were done using model species, whereas a few successful studies addressed the issue using non-model species [11–14]. Clearly, further studies are required to better understand the genetic causes for diverse ecologically important trait changes in the context of the tempo and mode of species range expansion.

In this paper, we focus on a wild wheat species *Aegilops tauschii* Coss. (formerly known as *Aegilops squarrosa* L.) and explore its potential for use for studies on ecologically important trait change during species range expansion. *Ae. tauschii* is a selfing diploid species that has a wide geographic range in central Eurasia with its center of distribution in the southern coastal region of the Caspian Sea and Azerbaijan. It is known as the D genome progenitor of hexaploid common wheat *Triticum aestivum* L., and much work has been done to clarify how the *Ae. tauschii* germplasm, a valuable genetic resource for wheat breeding, is structured [15–25].

Apart from its agronomic importance, *Ae. tauschii* has intriguing ecological and biogeographic features. The species is highly polymorphic in morphology and has a wide ecological amplitude: its natural habitats occur in sandy seashores, margins of deserts, stony hills, steppes, wastelands, roadsides and humid temperate forests within a vast range that spreads eastward to western China via the Kopet Dag Mountains of Turkmenistan and westward to central Syria via the valleys of southeastern Turkey. Weedy stands of *Ae. tauschii* are often found in wheat and barley fields within the range [26–28]. Furthermore, *Ae. tauschii* has a distinctive pattern of geographic distribution relative to other species of the same genus. According to a modern monograph, the genus *Aegilops* has 10 diploid and 12 polyploid species. The diploid species are thought to have radiated some million years ago in the Transcaucasus [27, 29, 30]. Interestingly, *Ae. tauschii* is the only diploid species that expanded its range eastward from this primary region of origin to the Asian part of the continent, whereas all the other diploid species migrated west- and southwestward. No natural stands of *Aegilops* species other than *Ae. tauschii* is currently known with certainty in China. Accordingly, *Ae. tauschii* may provide a suitable material for studies on ecologically important trait changes during range expansion, because the species appears to have expanded its natural range to Asia mainly through adaptation to a wide array of environmental conditions of the continent.

The patterns of genetic variations have been studied in *Ae. tauschii* at the molecular level, but relatively little is known about how the species’ phenotypic natural variations for ecologically important traits is structured. Previously, we analyzed the geographic and phylogenetic patterns of flowering time and spikelet shape variations and showed that the chloroplast-DNA-haplotype-defined intraspecific lineages that contain the Asian populations tend to have the early-flowering and small-spikelet-size phenotypes [31–33]. In this paper, we attempt to clarify the intraspecific patterns of changes in key functional life history and phenological traits during the eastward species range expansion using a nuclear-DNA-polymorphism-based approach [22–24]. Our comparative analyses of the patterns of natural variations for those traits and their association with the intraspecific lineage structure showed that (1) the eastward expansion to Asia was driven by an intraspecific sublineage (named TauL1b), (2) high seed production ability likely had an important role at the initial dispersal stage of TauL1b’s expansion to Asia, and (3) the phenological change to early flowering habit was one of the key adaptation events for TauL1b to further expand its range in Asia. These findings suggest that the current *Ae. tauschii*’s range is the product of complex evolutionary processes that involve changes in both life history and phenological traits. On the basis of these findings, we concluded that the genetic and ecological details of how the *Ae. tauschii* range formed deserve further studies.

## Results

### Intraspecific lineage structure in *Ae. tauschii*

**Fig. 1:**
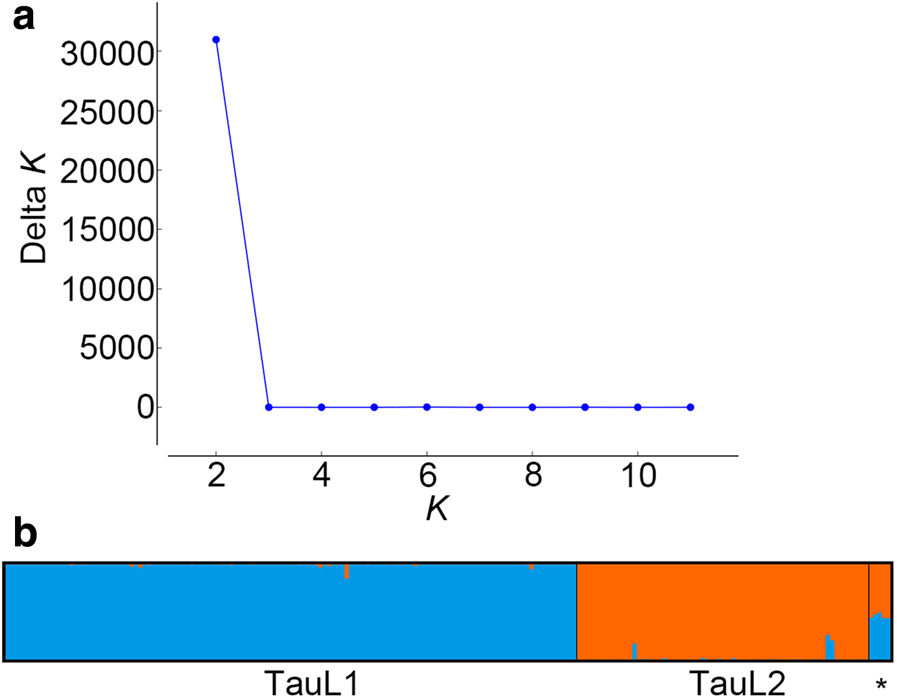
STRUCTURE analysis of the 206 *Ae. tauschii* accessions. **a** Plot of Δ*K.* **b** Proportional membership (*Q*) of each accession at *K* = 2. The asterisk denotes TauL3

In our previous study that used Diversity Arrays Technology (DArT) to genotype 206 *Ae. tauschii* accessions with 169 DArT markers, three intraspecific lineages, named TauL1, TauL2, and TauL3, were found [23]. These lineages have contrasting patterns of geographic distributions: the TauL1 accessions are widely spread across the species range, whereas the TauL2 and TauL3 accessions are restricted to the Transcaucasus/Middle East region and Georgia, respectively. To further investigate the intraspecific lineage structure, we analyzed a larger DArT genotype dataset that was obtained by the use of an additional 84 markers (253 markers in total) for the same set of *Ae. tauschii* accessions using a Bayesian clustering approach implemented in the software program STRUCTURE 2.3 [34]. Calculation of Δ*K*, an ad hoc statistic based on the second order rate of change of likelihood of *K* (the number of putative genepools that best explained the pattern of variations at the DArT-marker loci) [35], based on the STRUCTURE output, indicated that the 206 accessions are grouped into two clusters: one being TauL1 and the other consisting of TauL2 and TauL3 (Fig. 1). Therefore, the small TauL3 lineage (having five accessions) might be related to TauL2, but we kept them separate in the following analyses, because the TauL3 accessions have distinctive chloroplast DNA haplotypes [31].

**Fig. 2:**
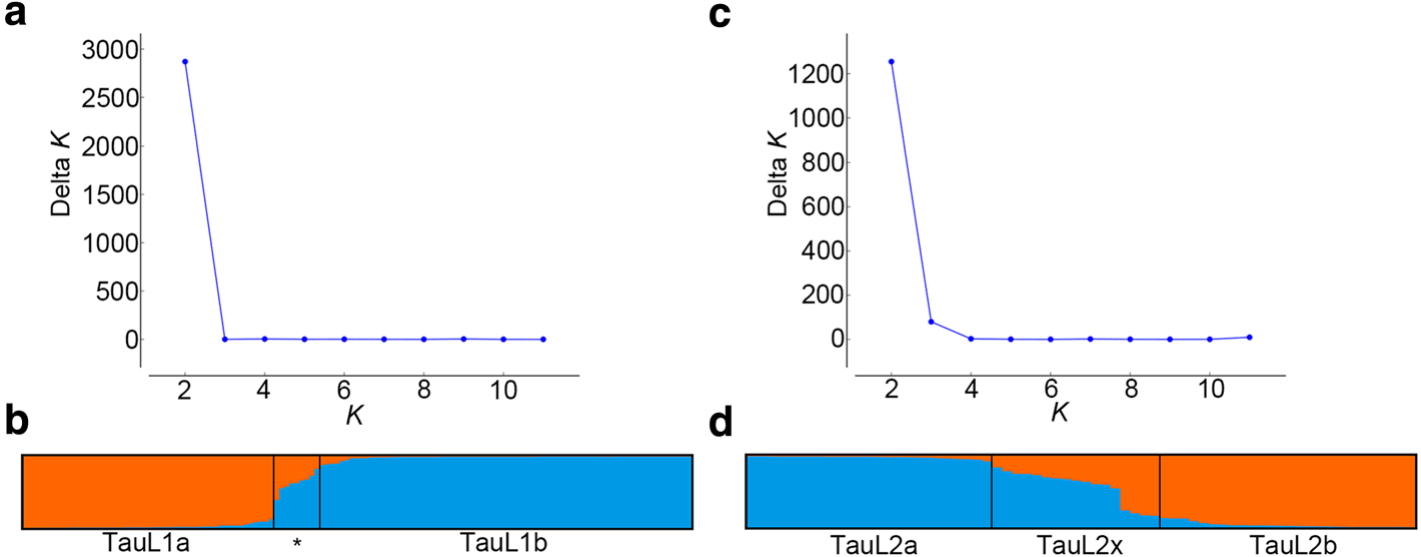
STRUCTURE analysis of the TauL1 and TauL2 lineages. **a** Plot of Δ*K* for the 133 TauL1 accessions. **b** Proportional membership (*Q*) of each TauL1 accession at *K* = 2. The asterisk denotes TauL1x. **c** Plot of Δ*K* for the 68 TauL2 accessions. **d** Proportional membership (*Q*) of each TauL2 accession at *K* = 2

A separate STRUCTURE analysis done for TauL1 (133 accessions) and TauL2 (68 accessions) indicated pronounced genepool differentiation at *K* = 2 within each lineage (Fig. 2). At *K* = 2, the 133 TauL1 accessions were classified into three sublineage groups: TauL1a (50 accessions), TauL1b (74 accessions), and the intermediates (named TauL1x) (9 accessions), using a threshold of 0.85 for the *Q* statistics (i.e., the estimated membership coefficients for each individual in each genepool). Similarly, the 68 TauL2 accessions were classified into three sublineage groups: TauL2a (26 accessions), TauL2b (25 accessions), and the intermediates (named TauL2x) (17 accessions) using the same *Q* statistics threshold.

**Fig. 3:**
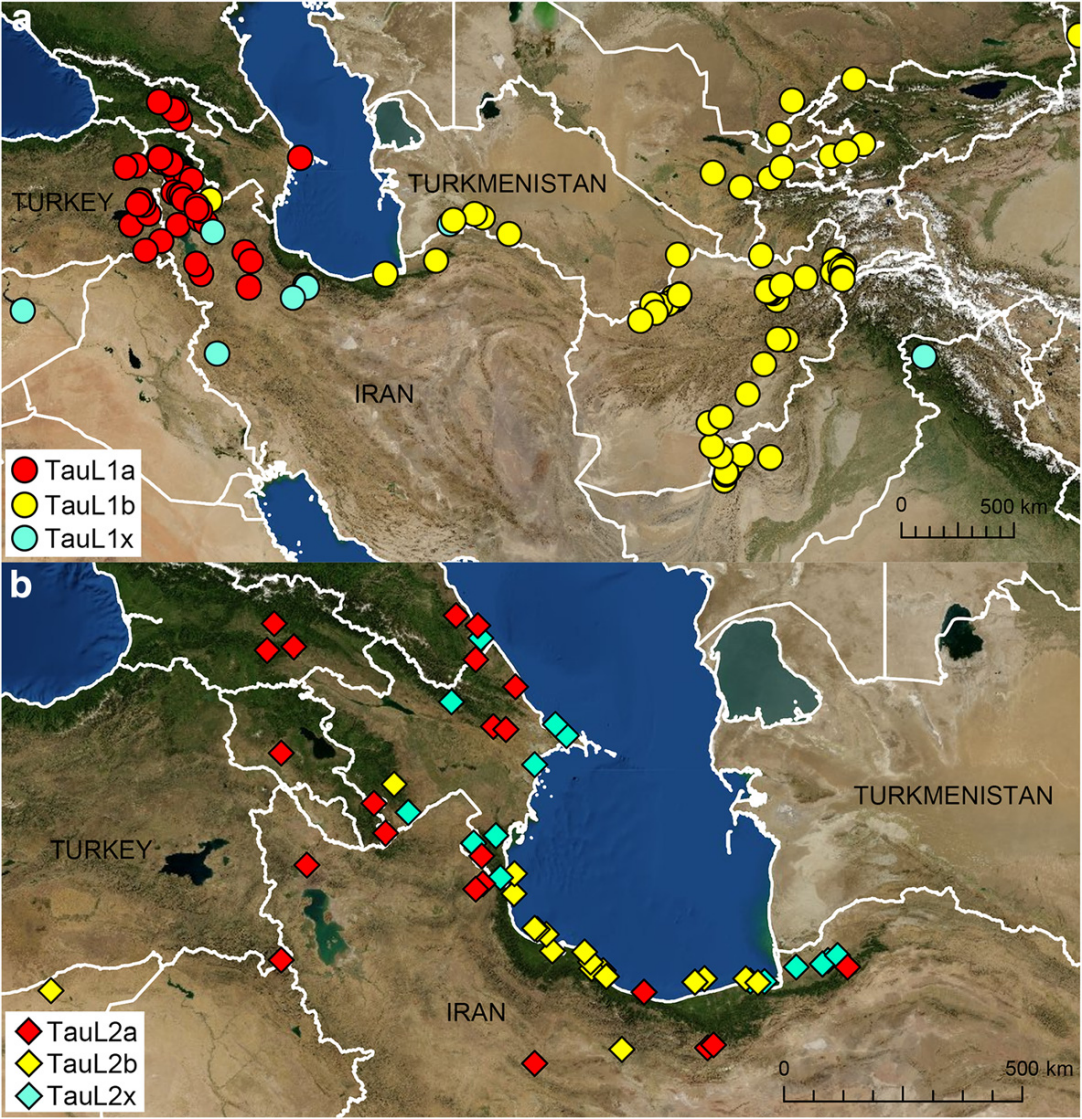
Geographic distributions of the TauL1 and TauL2 sublineages. **a** The TauL1 sublineages. TauL1a, TauL1b, and TauL1x are colored *red*, *yellow*, and *blue*, respectively. The six accessions representing adventive populations in the Shaanxi and Henan provinces are not shown. **b** The TauL2 sublineages. TauL2a. TauL2b, and TauL2x are colored red, yellow and blue, respectively

These sublineages markedly differ in their patterns of geographic distributions (Fig. 3). TauL1b was found to be the only sublineage that occurs in the Asian part of *Ae. tauschii*’s natural range (Turkmenistan and the countries to its east). The TauL1b accessions only rarely occur outside Asia, whereas the natural distribution of the TauL1a accessions is restricted to the Transcaucasus and Middle East (Iran and the countries to its west). Interestingly, one (AT 47) of the six accessions of the adventive populations in central China belongs to the TauL1a lineage, but the rest five (AT 55, AT 60, AT 76, AT80, and PI 508264) to the TauL1b lineage. The chloroplast DNA haplotype evidence supported that TauL1a is ancestral to TauL1b: all but one TauL1a accessions belonged to the ancestral chloroplast DNA haplogroup lineage HGL7, whereas most TauL1b accessions (53 out of the 74 accessions) belonged to the derived chloroplast DNA haplogroup lineage HGL16 (Additional file 1: Table S1) [31]. Both TauL2a and TauL2b are endemic to the Transcaucasus and Middle East: the TauL2a accessions tend to spread in the Transcaucasus, whereas TauL2b has its distribution center in the Caspian coastal region. The TauL1x accessions mostly fringes the southern limit of the species’ range and the TauL2x accessions tends to occur in the Caspian coastal region, but the distribution patterns of these intermediate accessions is not clear. The overall patterns of sublineage geographic distributions are consistent with the results of previous studies that used distance-based clustering approaches [22, 24]. All these findings show that *Ae. tauschii*’s eastward range expansion to Asia was driven by the TauL1b sublineage.

### Reproductive trait variation patterns and their association with the intraspecific lineage structure

To address how the TauL1b accessions are distinctive with respect to ecologically important traits, we compared the natural phenotypic variation patterns of seed production and flowering time traits between the intraspecific groups. A seed production trait dataset, which contained the measurements/counts of spike length, numbers of spikelets per spike, anther length, anther width, ovary length, and ovary width, was obtained for 198 DArT-genotyped accessions that were grown using a common garden experimental design. Of these traits, spike length and numbers of spikelets per spike were the indicators of seed production ability. Anther and ovary sizes (length and width) were included as the measures of resource allocation that may be associated with seed production through a possible trade-off, i.e., setting many seeds at the expense of anther and ovary development. In fact, varying degrees of negative correlation were observed between spikelet length and anther and ovary lengths and between numbers of spikelets per spike and anther and ovary lengths, suggesting that the accessions having small anthers and ovaries may have the potential to set a larger numbers of small seeds through efficient selfing which requires smaller amounts of pollen (Table 1). Flower manipulation experiments done in *Nigella sativa* L. showed the existence of reproductive trade-offs between stamen development and flower bud number [36]. Anther length represents the key trait in pollination ecology of *Ae. tauschii* [37]. A common-garden-experiment-based flowering time dataset for the same 198 accessions was taken from [31].

**Table 1:** Correlation coefficients* between seed production traits and their *p-*values

Anther/ovary trait	Spike length	*p-*value	Number of spikelet per spike	*p-*value
Anther length	−0.35	<0.0001	−0.61	<0.0001
Anther width	−0.18	0.013	−0.38	<0.0001
Ovary length	−0.14	0.043	−0.28	<0.0001
Ovary width	−0.20	0.005	−0.32	<0.0001

*The coefficient of correlation between spike length and number of spikelet per spike is 0.81 (*p* < 0.0001)

**Fig. 4:**
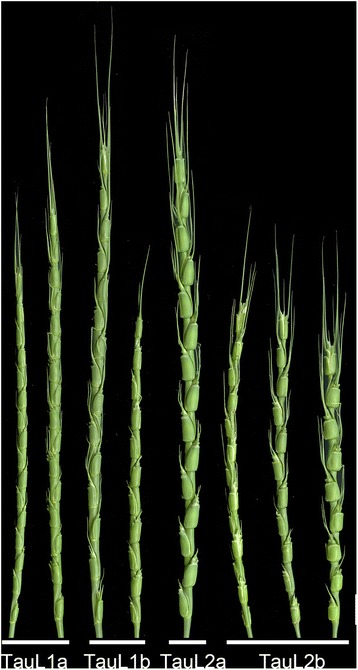
Early spikes of *Ae. tauschii* accessions. The accession names are IG 48747 (seed-production PC value = 3.51), KU-2824 (1.49), IG 48567 (0.48), KU-2008 (−0.27), KU-20-8 (−4.08), IG 47202 (−0.20), KU-20-10 (−2.29), and KU-2091 (−3.18) (from left to right). *Bar* 1 cm

To simplify analysis, we performed a principal component analysis of the seed production traits and combined them into a single virtual trait (i.e., the first principal component) that defined the axis of maximum phenotypic divergence among the accessions in a multivariate space. The first principal component (PC1) explained 53.1 % of the total variance. The eigenvectors for the PC1 were −0.33 (spike length), −0.43 (numbers of spikelets per spike), 0.42 (anther length), 0.43 (anther width), 0.41 (ovary length), and 0.43 (ovary width) (Table 2). We defined the sign-reversed PC1 as the seed-production PC. Accordingly, the seed-production PC values provided useful phenotype indexes: as they changed from low to high, spikelet length and numbers of spikelets per spike changed from short and few to long and many, whereas anther and ovary sizes changed from long and wide to short and narrow (Fig. 4). At the species level, no obvious geographic cline was observed for the seed-production PC values (Additional file 2: Figure S1).

**Table 2:** Eigenvectors for the first, second and third principal components^a^

Trait	PC1	PC2	PC3
Spike length	−0.33	0.58	0.40
Number of spikelet per spike	−0.43	0.48	0.08
Anther length	0.42	−0.14	0.63
Anther width	0.43	0.24	0.44
Ovary length	0.41	0.43	−0.35
Ovary width	0.43	0.41	−0.35

^a^The first PC (PC1), the second PC (PC2), and the third PC (PC3) explain 53.1, 23.9, and 12.1 % of the total variance, respectively

**Fig. 5:**
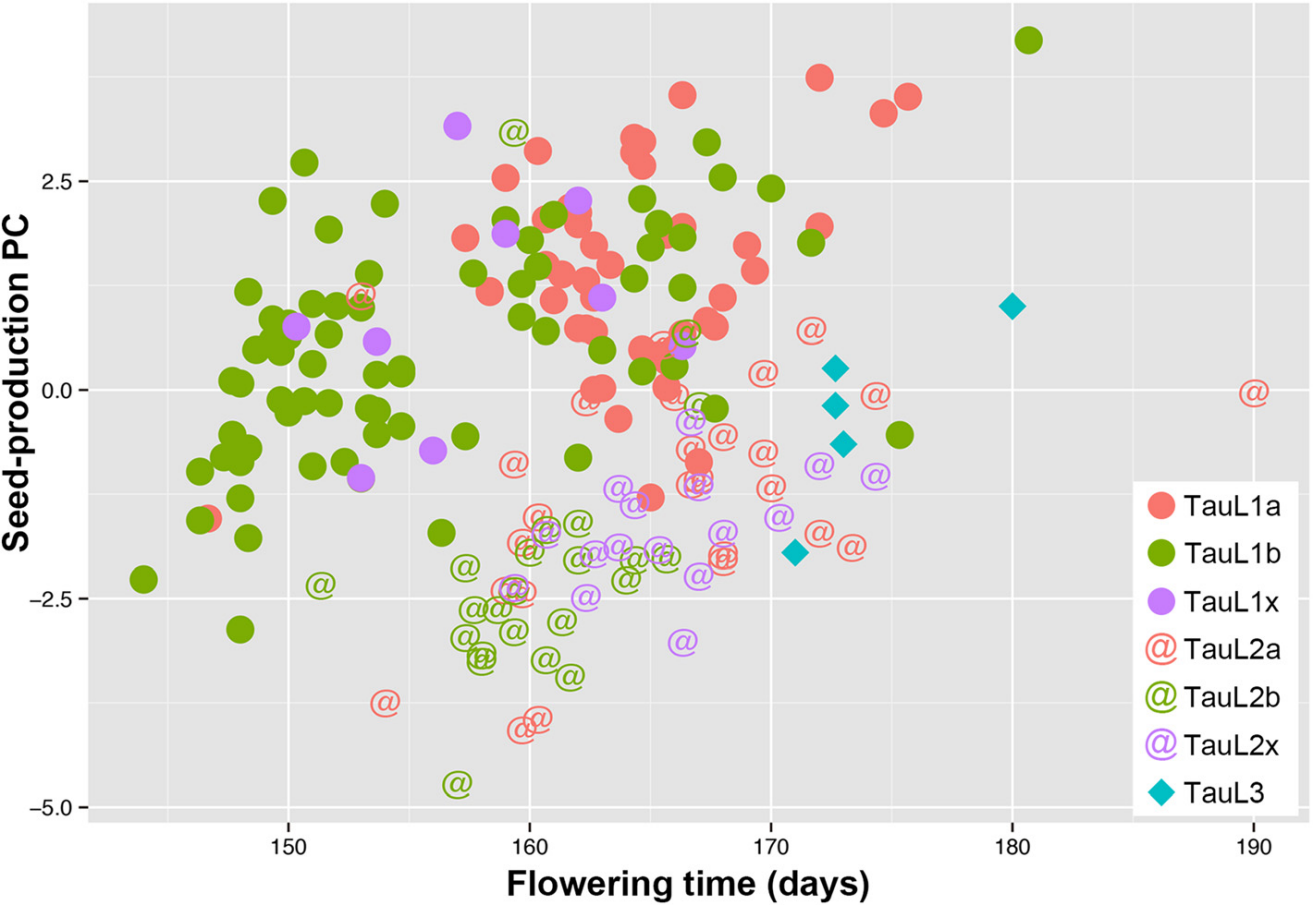
Graph of flowering time (*x*) and seed-production PC (*y*). Circles, at signs, and diamonds denote the TauL1, TauL2, and TauL3 lineages, respectively. Within each TauL1 and TauL2, pink indicates the presumable ancestral sublineage (TauL1a or TauL2a), whereas green the derived sublineage (TauL1b or TauL2b). The TauL1x and TauL2x accessions are colored purple

We plotted the 198 accessions in a graph of flowering time against seed-production PC (Fig. 5). The group-wise means of flowering time ranged from 155.7 days (TauL1b) to 173.9 days (TauL3) (Table 3). The TauL1b sublineage had many early-flowering accessions (less than 157.0 days to flower, 45 accessions), whereas the accessions of other groups had intermediate-flowering (157.0 days ≤ flowering time < 169.0 days) and late-flowering (169.0 days ≤ flowering time) phenotypes with a few exceptions. The group-wise means of seed-production PC values ranged from 1.36 (TauL1a) to −2.12 (TauL2b) (Table 3). The group-wise means of seed-production PC values were significantly larger for the TauL1 sublineages/group than for the TauL2 sublineages/group, indicating that the TauL1 accessions are characteristic in that they have small anthers and ovaries and set many seeds on long spikes (Table 3). Within the TauL1 lineage, many TauL1a accessions had large seed-production PC values relative to the TauL1b accessions (Fig. 5), but the difference between the means was not significant (Table 3). The TauL2 accessions had relatively small seed-production PC values, indicating that they tended to have large anthers and ovaries and set small numbers of seeds on short spikes. The TauL3 accessions were phenotypically intermediate between TauL2 and TauL1. These results clearly showed that the reproductive trait variation patterns are associated with the intraspecific lineage structure to a considerable extent and that the early flowering phenotypes primarily characterize the TauL1b accessions.

**Table 3:** Mean seed-production PC and flowering time for the *Ae. tauschii* lineage/sublineages/groups

Seed-production PC			Flowering time (days)		
Lineage/sublineage/group	N	Mean*	Lineage/sublineage/group	N	Mean**
TauL1a	48	1.36^a^	TauL1b	71	155.7^a^
TauL1x	9	0.94^ab^	TauL1x	9	157.8
TauL1b	71	0.50^ab^	TauL2b	23	160.4^b^
TauL3	5	−0.31^bc^	TauL1a	48	164.2^c^
TauL2a	26	−1.22^cd^	TauL2x	16	165.9^c^
TauL2x	16	−1.69^cd^	TauL2a	26	165.9^c^
TauL2b	23	−2.12^d^	TauL3	5	173.9^d^

*Means sharing a common superscript are not significantly different (Tukey-Kramer test, *p* < 0.05)

**Means sharing a common superscript are not significantly different (Dunnett’s T3 test, *p* < 0.05). The test was done excluding the mean of TauL1x because of the lack of statistical power

**Fig. 6:**
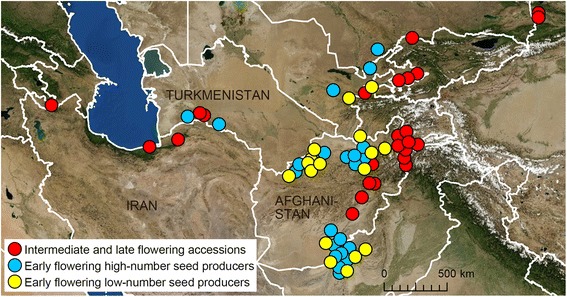
Geographic distributions of the TauL1b accessions. The *red*, *blue*, and *yellow circles* denote the intermediate and late flowering accessions, early flowering high-number seed producers, and early flowering low-number seed producers, respectively. The six accessions representing adventive populations in the Shaanxi and Henan provinces, all of which are early flowering low-number seed producers, are not shown

 In the graph, the TauL1b’s intermediate and late flowering accessions largely overlapped with the TauL1a accessions, but the early flowering accessions did not with an exception of a Chinese accession AT47 (flowering time = 146.7 days) (Fig. 5). The mean flowering time of TauL1b (155.7 days) was significantly smaller than that of TauL1a (164.2 days) (Table 3). This indicated that the TauL1b’s intermediate and late flowering accessions are comparable to the TauL1a accessions in their seed production traits, whereas the early flowering accessions are distinctive. Furthermore, the early flowering accessions had diverse seed production phenotypes that could be divided into two groups: early flowering high-number seed producers (seed-production PC values > 0) and early flowering low-number seed producers (seed-production PC values ≤ 0). The geographic distribution of the intermediate and late flowering accessions and the early flowering accessions (both high-number seed producers and low-number seed producers) widely overlaps, but the intermediate and late flowering accessions tend to reside in the north of the TauL1b range, whereas the early flowering accessions in the south (Fig. 6).

**Fig. 7:**
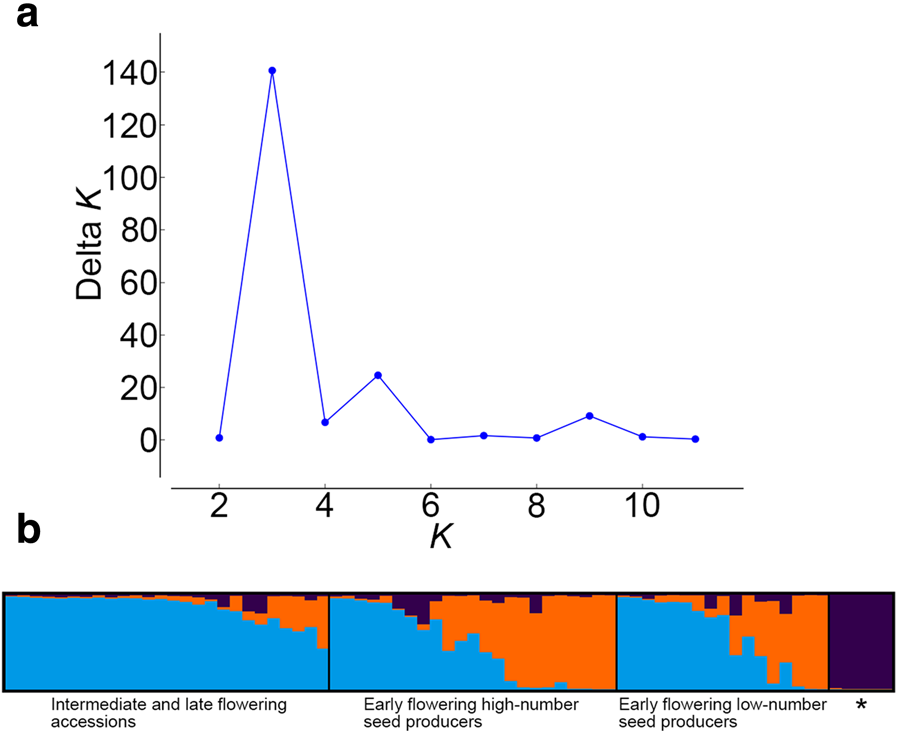
STRUCTURE analysis of the 74 TauL1b accessions. **a** Plot of Δ*K*. **a** Proportional membership (*Q*) of each TauL1b accession at *K* = 3. The asterisk denotes the adventive Chinese accessions

To examine if the TauL1b’s early flowering accessions are genetically differentiated from the intermediate and late flowering accessions, we further analyzed the genepool structure of the TauL1b sublineage. A STRUCTURE analysis done by the use of 117 polymorphic DArT markers showed that the TauL1b sublineage consists of two large and one small genepools (Fig. 7). The majority of the alleles of the intermediate and late flowering accessions were derived from one of the large genepools. Roughly, half of the alleles of the early accessions (excluding the adventive Chinese accessions) were derived from the same large genepool, whereas the other large genepool was the major source for the rest of the alleles. Pairwise *F*_st_ values showed low but significant degrees of genetic differentiation between the early flowering high-number seed producers and the intermediate and late flowering accessions (*F*_st_ = 0.069) and between the early flowering low-number seed producers and the late flowering accessions (*F*_st_ = 0.057) (Table 4). The adventive Chinese accession alleles were almost exclusively originated from the small genepool. These results indicated that the TauL1b early flowering accessions were genetically differentiated from the intermediate and late flowering accessions, whereas their genetic composition is relatively complex.

**Table 4:** Pairwise *F*_st_ values between the TauL1b phenotype groups^a^

Phenotype group	Intermedate and late flowering accessions	Early flowering high-number seed producers	Early flowering low-number seed producers
Intermedate and late flowering accessions		0.001	0.001
Early flowering high-number seed producers	0.069		0.607
Early flowering low-number seed producers	0.057	0.024	

^a^ *F*_st_ values are below the diagonal. Permutation *p*-values based on 999 permutations are shown above the diagonal

## Discussion

In this study, the TauL1b sublineage was identified as the driver for the *Ae. tauschii*’s eastward range expansion. Furthermore, the comparative analysis of the reproductive trait variation patterns and their association with intraspecific lineage structure provided some insights into the roles of the seed production and flowering time traits in range expansion.

The seed production traits likely had an important role in the initial stage of dispersal from Middle East to Asia. This inference is based on the facts that the TauL1 accessions that tend to have small anthers and ovaries and set many seeds on long spikes made their way to Asia, but the TauL2 accessions that tend to have large anthers and ovaries and set small numbers of seeds on short spikes did not. High seed production ability, therefore, may have been one of the phenotypes that facilitated the TauL1’s long distance dispersal from Middle East to Asia. In fact, seed number is a key trait in realizing long distance dispersal through animal guts in grasslands [38]. For this reason, we speculate that grazing mammals might have served as vectors in the TauL1’s eastward dispersal. To better support this view, further comparative analyses that incorporate the total number of seeds that each *Ae. tauschii* accession sets per plant are required. Each TauL1a and TauL2a has the center of distribution in the Transcaucasus and represents ancestral sublineages of the species. The phenotypic divergence in the seed production traits probably has a deep evolutionary origin, because the difference between the TauL1a and TauL2a sublineages in the mean seed-production PC values is statistically significant (Table 3).

Flowering time change likely contributed to the TauL1b accessions’ adaptation to the Asian environment. In the TauL1b sublineage, 45 out of the 71 accessions (excluding three accessions for which the flowering time data are missing) had the early flowering phenotype, whereas the remaining 26 accessions were intermediate and late flowering. This is in stark contrast with the cases of other groups (i.e., TauL1a, TauL1x, TauL2a, TauL2b, and TauL3) in which the vast majority of the accessions were intermediate and late flowering with a few exceptions (Fig. 5). Furthermore, no early flowering TauL1a accession (except for a Chinese accession AT47) was found in this study, suggesting that the early flowering phenotype is not adaptive in the Transcaucasus and Middle East. One possible explanation for this observation is that the ancestral TauL1b accessions that first migrated to Asia had the intermediate and late flowering phenotypes together with the high-number seed producer phenotypes and that the early flowering accessions evolved from the ancestral accessions through adaptation to the Asian environment. The adaptive change to the early flowering phenotypes may have occurred in response to winter temperature variability and facilitated the TauL1b’s further range expansion in Asia [31]. In the TauL1b sublineage, many early flowering accessions had lowered seed-production PC values relative to the intermediate and late flowering accessions (Fig. 5). This may suggest that, in the early flowering accessions, the shortened growth periods caused reduction in seed production.

## Conclusions

This study provides for the first time a broad picture of the process of *Ae. tauschii*’s eastward range expansion in which life history (i.e., seed production) and phenological (i.e., flowering time) traits may have had respective roles in the dispersal to and adaptation in Asia. The existence of a major intraspecific lineage (TauL1) that had the potential to produce long-traveling seeds and TauL1’s possession of alleles that caused the adaptive flowering time evolution through mutation appear to have contributed to realizing the cross-continent range expansion in *Ae. tauschii*. Most likely, long-standing and later arisen phenotypic variations of various ecologically important traits opened avenues for the expansion. In another *Aegilops* species, *Aegilops triuncialis* (L.) Á. Löve, adaptation to serpentine soils was shown to have facilitated the species’ recent rapid invasion into California, USA [39]. Therefore, roles of ecologically important traits other than seed production and flowering time, such as edaphic preference, osmotic stress tolerance, and disease resistance, in *Ae. tauschii*’s range expansion must further be studied. In such studies, spatial pattern analyses of phenotypic variations and their relationships with abiotic variables would be of great interest, because environmental factors could influence the phenotypic landscapes of ecologically important traits that may have roles in species range expansion [40].

Since Darwin’s proposal, ecologists have long assumed that species having large ecological amplitude show great phenotypic variation [41]. Our analyses captured a glimpse of such phenotypic diversification that took place in the course of *Ae. tauschii*’s range expansion: the seed production trait divergence in the ancestral lineages, the rise of the high-number seed producer phenotype, and the dispersal to Asia, a brand-new stage for flowering time diversification through adaptive changes in response to local conditions. The clear association of seed production and flowering time patterns with the intraspecific lineage divergence described in this study invites further genetic research to understand the mechanisms underlying the changes in these key functional traits during range expansion.

## Methods

### Plant materials

Two hundred *Ae. tauschii* Coss. accessions representing the entire natural habitat range and six accessions (AT 47, AT 55, AT 60, AT 76, AT 80, and PI 508264) representing adventive populations in the Shaanxi and Henan provinces of China were used (Additional file 1: Table S1).

### DArT marker genotyping

The DArT marker genotyping was done at Diversity Arrays Technology Pty. Ltd., Yarralumla, Australia, using the array that was developed for *Ae. tauschii* genotyping [42]. Of the several thousand markers that showed polymorphisms between the accessions, 253 were selected based on the data quality, redundancy, and reproducibility. The map information provided by Diversity Arrays Technology Pty. Ltd. was used to infer the distribution of the marker loci that spread across the seven chromosomes of *Ae. tauschii*. The genotype data for 19 *Ae. tauschii* accessions (AT 47, AT 76, CGN 10734, CGN 10768, IG126991, IG127015, IG 47202, IG 47203, IG 49095, KU-2022, KU-2035, KU-2063, KU-2069, KU-2097, KU-2109, KU-2136, KU-2159, KU-2809, and KU-2814) were obtained from [43]. The genotypes at each locus were scored as either presence (coded as “1”) or absence (coded as “0”) of hybridization to the corresponding array element. For the full 206 accessions by 253 markers dataset, the missing data percentages ranged from 0 to 6.3 % (mean 1.7 %) between accessions and 0 to 11.7 % (mean 1.7 %) between markers.

### Bayesian clustering

The STRUCTURE software [34] was used for Bayesian clustering. Because *Ae. tauschii* is a highly selfing species and largely homozygous, this analysis was done using a haploid setting. The algorithm was run with a burn-in length of 500,000 and then 500,000 Markov Chain Monte Carlo simulations for estimating parameters. To find the best *K*, we performed 10 independent runs for each *K* between 1 and 12 using the admixture model and correlated allele frequencies. Each accession was assigned to a sublineage or genepool based on the *Q* statistics that were calculated through 100 independent runs for the best *K*. We used the STRUCTURE HARVESTER software [44] to calculate the Evanno’s ∆*K* values and the CLUMPP algorithm [45] implemented in the CLUMPAK software [46] to combine the outputs from the STRUCTURE software with the Greedy search option (2000 repeats).

### Common garden experiment

The *Ae. tauschii* plants were grown under field conditions at Kobe University. For each accession, three seeds propagated by selfing from a single plant were sown. We arranged the accessions in the field using a randomized design and chose a single healthy individual for measuring traits. Spike lengths and numbers of spikelets per spike were measured/counted using the first, second, and third spikes before anthesis. For anther and ovary measurements, only the components of the first and second florets of the central spikelets of the first, second, and third spikes before anthesis were used; for each accession, 18 anther and 6 ovary samples were measured. The mean of the replicated measurements over three spikes was calculated for each trait and used as the trait value in subsequent analyses.

### Statistic analysis

Statistical calculations were done with JMP software ver. 11.2 (SAS Institute) and SPSS software ver. 20 (IBM). The among-accession correlation matrix was used in the principal component analysis. GenAlEx ver. 6.5 was used to calculate pairwise *F*_st_ values [47].

## Additional files

Additional file 1: Table S1. The *Ae. tauschii* accessions used. (DOCX 31 kb) Additional file 2: Figure S1. Plot of the 198 *Ae. tauschii* accession geographic coordinates against the seed-production PC values. A. Latitude. B. Longitude. (TIFF 32105 kb)

## Abbreviation

DArT: Diversity arrays technology
