# Supplementary material for: Intraspecific lineage divergence and its association with reproductive trait change during species range expansion in central Eurasian wild wheat Aegilops tauschii Coss. (Poaceae)
Source: BMC Evol Biol. 2015 Sep 30;15:213. doi: 10.1186/s12862-015-0496-9 (PMC4589133; doi:10.1186/s12862-015-0496-9)
Supplement: Additional file 1: Table S1. — The Ae. tauschii accessions used. (DOCX 31 kb) [file 12862_2015_496_MOESM1_ESM.docx]

**Additional file 1: Table S1. The *Ae. tauschii* accessions used.** Source codes are IPK for Institut für Pflanzengenetik und Kulturpflanzenforschung, CGN for Centre for Genetic Resources, The Netherlands, ICARDA for International Center for Agricultural Research in the Dry Areas, KYOTO for Plant Germ-plasm Institute of Kyoto University, NBRP for National BioResources Project, OKAYAMA for Dr. Kenji Kato, Okayama University, and USDA for US Department of Agriculture.

| **No.** | **Species** | **Accession** | **Origin** | **Source** | **Chloroplast haplogroup lineage [31]** | **Lineage/sublinegae/group (this study)** |
| --- | --- | --- | --- | --- | --- | --- |
| 1 | *Aegilops tauschii* Coss. | AE 933 | Georgia | IPK | HGL7 | TauL1a |
| 2 | *Aegilops tauschii* Coss. | CGN 10734 | Armenia | CGN | HGL7 | TauL1a |
| 3 | *Aegilops tauschii* Coss. | IG 126273 | Armenia | ICARDA | HGL7 | TauL1a |
| 4 | *Aegilops tauschii* Coss. | IG 126280 | Armenia | ICARDA | HGL7 | TauL1a |
| 5 | *Aegilops tauschii* Coss. | IG 126293 | Armenia | ICARDA | HGL7 | TauL1a |
| 6 | *Aegilops tauschii* Coss. | IG 126353 | Armenia | ICARDA | HGL7 | TauL1a |
| 7 | *Aegilops tauschii* Coss. | IG 47196 | Azerbaijan | ICARDA | HGL7 | TauL1a |
| 8 | *Aegilops tauschii* Coss. | IG 48747 | Armenia | ICARDA | HGL7 | TauL1a |
| 9 | *Aegilops tauschii* Coss. | IG 48748 | Armenia | ICARDA | HGL7 | TauL1a |
| 10 | *Aegilops tauschii* Coss. | IG 48758 | Armenia | ICARDA | HGL7 | TauL1a |
| 11 | *Aegilops tauschii* Coss. | IG 49095 | Iran | ICARDA | HGL7 | TauL1a |
| 12 | *Aegilops tauschii* Coss. | KU-2113 | Iran | KYOTO/NBRP | HGL7 | TauL1a |
| 13 | *Aegilops tauschii* Coss. | KU-2115 | Iran | KYOTO/NBRP | HGL7 | TauL1a |
| 14 | *Aegilops tauschii* Coss. | KU-2116 | Iran | KYOTO/NBRP | HGL7 | TauL1a |
| 15 | *Aegilops tauschii* Coss. | KU-2120 | Iran | KYOTO/NBRP | HGL7 | TauL1a |
| 16 | *Aegilops tauschii* Coss. | KU-2121 | Iran | KYOTO/NBRP | HGL7 | TauL1a |
| 17 | *Aegilops tauschii* Coss. | KU-2131 | Turkey | KYOTO/NBRP | HGL7 | TauL1a |
| 18 | *Aegilops tauschii* Coss. | KU-2132 | Turkey | KYOTO/NBRP | HGL7 | TauL1a |
| 19 | *Aegilops tauschii* Coss. | KU-2133 | Turkey | KYOTO/NBRP | HGL7 | TauL1a |
| 20 | *Aegilops tauschii* Coss. | KU-2136 | Turkey | KYOTO/NBRP | HGL7 | TauL1a |
| 21 | *Aegilops tauschii* Coss. | KU-2137 | Turkey | KYOTO/NBRP | HGL7 | TauL1a |
| 22 | *Aegilops tauschii* Coss. | KU-2138 | Turkey | KYOTO/NBRP | HGL7 | TauL1a |
| 23 | *Aegilops tauschii* Coss. | KU-2140 | Turkey | KYOTO/NBRP | HGL7 | TauL1a |
| 24 | *Aegilops tauschii* Coss. | KU-2141 | Turkey | KYOTO/NBRP | HGL7 | TauL1a |
| 25 | *Aegilops tauschii* Coss. | KU-2142 | Iran | KYOTO/NBRP | HGL7 | TauL1a |
| 26 | *Aegilops tauschii* Coss. | KU-2143 | Iran | KYOTO/NBRP | HGL7 | TauL1a |
| 27 | *Aegilops tauschii* Coss. | KU-2144 | Iran | KYOTO/NBRP | HGL7 | TauL1a |
| 28 | *Aegilops tauschii* Coss. | KU-2145 | Iran | KYOTO/NBRP | HGL7 | TauL1a |
| 29 | *Aegilops tauschii* Coss. | KU-2148 | Iran | KYOTO/NBRP | HGL7 | TauL1a |
| 30 | *Aegilops tauschii* Coss. | KU-2149 | Iran | KYOTO/NBRP | HGL7 | TauL1a |
| 31 | *Aegilops tauschii* Coss. | KU-2150 | Iran | KYOTO/NBRP | HGL7 | TauL1a |
| 32 | *Aegilops tauschii* Coss. | KU-2151 | Iran | KYOTO/NBRP | HGL7 | TauL1a |
| 33 | *Aegilops tauschii* Coss. | KU-2152 | Iran | KYOTO/NBRP | HGL7 | TauL1a |
| 34 | *Aegilops tauschii* Coss. | KU-2809 | Armenia | KYOTO/NBRP | HGL7 | TauL1a |
| 35 | *Aegilops tauschii* Coss. | KU-2810 | Armenia | KYOTO/NBRP | HGL7 | TauL1a |
| 36 | *Aegilops tauschii* Coss. | KU-2814 | Armenia | KYOTO/NBRP | HGL7 | TauL1a |
| 37 | *Aegilops tauschii* Coss. | KU-2816 | Armenia | KYOTO/NBRP | HGL7 | TauL1a |
| 38 | *Aegilops tauschii* Coss. | KU-2821 | Armenia | KYOTO/NBRP | HGL7 | TauL1a |
| 39 | *Aegilops tauschii* Coss. | KU-2822A | Armenia | KYOTO/NBRP | HGL7 | TauL1a |
| 40 | *Aegilops tauschii* Coss. | KU-2823 | Armenia | KYOTO/NBRP | HGL7 | TauL1a |
| 41 | *Aegilops tauschii* Coss. | KU-2824 | Armenia | KYOTO/NBRP | HGL7 | TauL1a |
| 42 | *Aegilops tauschii* Coss. | KU-2826 | Georgia | KYOTO/NBRP | HGL7 | TauL1a |
| 43 | *Aegilops tauschii* Coss. | KU-2828 | Georgia | KYOTO/NBRP | HGL7 | TauL1a |
| 44 | *Aegilops tauschii* Coss. | KU-2834 | Georgia | KYOTO/NBRP | HGL7 | TauL1a |
| 45 | *Aegilops tauschii* Coss. | KU-2836 | Georgia | KYOTO/NBRP | HGL7 | TauL1a |
| 46 | *Aegilops tauschii* Coss. | PI 486270 | Turkey | USDA | HGL7 | TauL1a |
| 47 | *Aegilops tauschii* Coss. | PI 486274 | Turkey | USDA | HGL7 | TauL1a |
| 48 | *Aegilops tauschii* Coss. | PI 486277 | Turkey | USDA | HGL7 | TauL1a |
| 49 | *Aegilops tauschii* Coss. | PI 554319 | Turkey | USDA | HGL7 | TauL1a |
| 50 | *Aegilops tauschii* Coss. | AT 47 | China | OKAYAMA | HGL16 | TauL1a |
| 51 | *Aegilops tauschii* Coss. | AE 1038 | Tajikistan | IPK | HGL7 | TauL1b |
| 52 | *Aegilops tauschii* Coss. | CGN 10767 | Pakistan | CGN | HGL7 | TauL1b |
| 53 | *Aegilops tauschii* Coss. | IG 126489 | Turkmenistan | ICARDA | HGL7 | TauL1b |
| 54 | *Aegilops tauschii* Coss. | IG 127015 | Armenia | ICARDA | HGL7 | TauL1b |
| 55 | *Aegilops tauschii* Coss. | IG 48554 | Tajikistan | ICARDA | HGL7 | TauL1b |
| 56 | *Aegilops tauschii* Coss. | IG 48564 | Tajikistan | ICARDA | HGL7 | TauL1b |
| 57 | *Aegilops tauschii* Coss. | KU-2010 | Afghanistan | KYOTO/NBRP | HGL7 | TauL1b |
| 58 | *Aegilops tauschii* Coss. | KU-2012 | Afghanistan | KYOTO/NBRP | HGL7 | TauL1b |
| 59 | *Aegilops tauschii* Coss. | KU-2016 | Afghanistan | KYOTO/NBRP | HGL7 | TauL1b |
| 60 | *Aegilops tauschii* Coss. | KU-2025 | Afghanistan | KYOTO/NBRP | HGL7 | TauL1b |
| 61 | *Aegilops tauschii* Coss. | KU-2027 | Afghanistan | KYOTO/NBRP | HGL7 | TauL1b |
| 62 | *Aegilops tauschii* Coss. | KU-2028 | Afghanistan | KYOTO/NBRP | HGL7 | TauL1b |
| 63 | *Aegilops tauschii* Coss. | KU-2032 | Afghanistan | KYOTO/NBRP | HGL7 | TauL1b |
| 64 | *Aegilops tauschii* Coss. | KU-2035 | Afghanistan | KYOTO/NBRP | HGL7 | TauL1b |
| 65 | *Aegilops tauschii* Coss. | KU-2042 | Afghanistan | KYOTO/NBRP | HGL7 | TauL1b |
| 66 | *Aegilops tauschii* Coss. | KU-2058 | Afghanistan | KYOTO/NBRP | HGL7 | TauL1b |
| 67 | *Aegilops tauschii* Coss. | KU-2617 | Afghanistan | KYOTO/NBRP | HGL7 | TauL1b |
| 68 | *Aegilops tauschii* Coss. | KU-2630 | Afghanistan | KYOTO/NBRP | HGL7 | TauL1b |
| 69 | *Aegilops tauschii* Coss. | KU-2633 | Afghanistan | KYOTO/NBRP | HGL7 | TauL1b |
| 70 | *Aegilops tauschii* Coss. | PI 499262 | China | USDA | HGL7 | TauL1b |
| 71 | *Aegilops tauschii* Coss. | PI 508262 | China | USDA | HGL7 | TauL1b |
| 72 | *Aegilops tauschii* Coss. | AE 1090 | Kazakhstan | IPK | HGL16 | TauL1b |
| 73 | *Aegilops tauschii* Coss. | AT 55 | China | OKAYAMA | HGL16 | TauL1b |
| 74 | *Aegilops tauschii* Coss. | AT 60 | China | OKAYAMA | HGL16 | TauL1b |
| 75 | *Aegilops tauschii* Coss. | AT 76 | China | OKAYAMA | HGL16 | TauL1b |
| 76 | *Aegilops tauschii* Coss. | AT 80 | China | OKAYAMA | HGL16 | TauL1b |
| 77 | *Aegilops tauschii* Coss. | CGN 10768 | Pakistan | CGN | HGL16 | TauL1b |
| 78 | *Aegilops tauschii* Coss. | CGN 10769 | Pakistan | CGN | HGL16 | TauL1b |
| 79 | *Aegilops tauschii* Coss. | CGN 10770 | Pakistan | CGN | HGL16 | TauL1b |
| 80 | *Aegilops tauschii* Coss. | CGN 10771 | Pakistan | CGN | HGL16 | TauL1b |
| 81 | *Aegilops tauschii* Coss. | IG 108561 | Pakistan | ICARDA | HGL16 | TauL1b |
| 82 | *Aegilops tauschii* Coss. | IG 120735 | Turkmenistan | ICARDA | HGL16 | TauL1b |
| 83 | *Aegilops tauschii* Coss. | IG 120736 | Uzbekistan | ICARDA | HGL16 | TauL1b |
| 84 | *Aegilops tauschii* Coss. | IG 123910 | Uzbekistan | ICARDA | HGL16 | TauL1b |
| 85 | *Aegilops tauschii* Coss. | IG 126387 | Turkmenistan | ICARDA | HGL16 | TauL1b |
| 86 | *Aegilops tauschii* Coss. | IG 131606 | Kyrgyzstan | ICARDA | HGL16 | TauL1b |
| 87 | *Aegilops tauschii* Coss. | IG 46663 | Pakistan | ICARDA | HGL16 | TauL1b |
| 88 | *Aegilops tauschii* Coss. | IG 46666 | Pakistan | ICARDA | HGL16 | TauL1b |
| 89 | *Aegilops tauschii* Coss. | IG 46682 | Pakistan | ICARDA | HGL16 | TauL1b |
| 90 | *Aegilops tauschii* Coss. | IG 48518 | Turkmenistan | ICARDA | HGL16 | TauL1b |
| 91 | *Aegilops tauschii* Coss. | IG 48539 | Uzbekistan | ICARDA | HGL16 | TauL1b |
| 92 | *Aegilops tauschii* Coss. | IG 48559 | Tajikistan | ICARDA | HGL16 | TauL1b |
| 93 | *Aegilops tauschii* Coss. | IG 48565 | Uzbekistan | ICARDA | HGL16 | TauL1b |
| 94 | *Aegilops tauschii* Coss. | IG 48567 | Uzbekistan | ICARDA | HGL16 | TauL1b |
| 95 | *Aegilops tauschii* Coss. | KU-20-6 | Pakistan | KYOTO/NBRP | HGL16 | TauL1b |
| 96 | *Aegilops tauschii* Coss. | KU-2001 | Pakistan | KYOTO/NBRP | HGL16 | TauL1b |
| 97 | *Aegilops tauschii* Coss. | KU-2003 | Pakistan | KYOTO/NBRP | HGL16 | TauL1b |
| 98 | *Aegilops tauschii* Coss. | KU-2006 | Pakistan | KYOTO/NBRP | HGL16 | TauL1b |
| 99 | *Aegilops tauschii* Coss. | KU-2008 | Pakistan | KYOTO/NBRP | HGL16 | TauL1b |
| 100 | *Aegilops tauschii* Coss. | KU-2018 | Afghanistan | KYOTO/NBRP | HGL16 | TauL1b |
| 101 | *Aegilops tauschii* Coss. | KU-2022 | Afghanistan | KYOTO/NBRP | HGL16 | TauL1b |
| 102 | *Aegilops tauschii* Coss. | KU-2039 | Afghanistan | KYOTO/NBRP | HGL16 | TauL1b |
| 103 | *Aegilops tauschii* Coss. | KU-2043 | Afghanistan | KYOTO/NBRP | HGL16 | TauL1b |
| 104 | *Aegilops tauschii* Coss. | KU-2044 | Afghanistan | KYOTO/NBRP | HGL16 | TauL1b |
| 105 | *Aegilops tauschii* Coss. | KU-2050 | Afghanistan | KYOTO/NBRP | HGL16 | TauL1b |
| 106 | *Aegilops tauschii* Coss. | KU-2051 | Afghanistan | KYOTO/NBRP | HGL16 | TauL1b |
| 107 | *Aegilops tauschii* Coss. | KU-2056 | Afghanistan | KYOTO/NBRP | HGL16 | TauL1b |
| 108 | *Aegilops tauschii* Coss. | KU-2059 | Afghanistan | KYOTO/NBRP | HGL16 | TauL1b |
| 109 | *Aegilops tauschii* Coss. | KU-2061 | Afghanistan | KYOTO/NBRP | HGL16 | TauL1b |
| 110 | *Aegilops tauschii* Coss. | KU-2063 | Afghanistan | KYOTO/NBRP | HGL16 | TauL1b |
| 111 | *Aegilops tauschii* Coss. | KU-2066 | Afghanistan | KYOTO/NBRP | HGL16 | TauL1b |
| 112 | *Aegilops tauschii* Coss. | KU-2082 | Iran | KYOTO/NBRP | HGL16 | TauL1b |
| 113 | *Aegilops tauschii* Coss. | KU-2087 | Iran | KYOTO/NBRP | HGL16 | TauL1b |
| 114 | *Aegilops tauschii* Coss. | KU-2612 | Afghanistan | KYOTO/NBRP | HGL16 | TauL1b |
| 115 | *Aegilops tauschii* Coss. | KU-2621 | Afghanistan | KYOTO/NBRP | HGL16 | TauL1b |
| 116 | *Aegilops tauschii* Coss. | KU-2624 | Afghanistan | KYOTO/NBRP | HGL16 | TauL1b |
| 117 | *Aegilops tauschii* Coss. | KU-2627 | Afghanistan | KYOTO/NBRP | HGL16 | TauL1b |
| 118 | *Aegilops tauschii* Coss. | KU-2632 | Afghanistan | KYOTO/NBRP | HGL16 | TauL1b |
| 119 | *Aegilops tauschii* Coss. | KU-2635 | Afghanistan | KYOTO/NBRP | HGL16 | TauL1b |
| 120 | *Aegilops tauschii* Coss. | KU-2636 | Afghanistan | KYOTO/NBRP | HGL16 | TauL1b |
| 121 | *Aegilops tauschii* Coss. | KU-2638 | Afghanistan | KYOTO/NBRP | HGL16 | TauL1b |
| 122 | *Aegilops tauschii* Coss. | KU-2639 | Afghanistan | KYOTO/NBRP | HGL16 | TauL1b |
| 123 | *Aegilops tauschii* Coss. | PI 476874 | Afghanistan | USDA | HGL16 | TauL1b |
| 124 | *Aegilops tauschii* Coss. | PI 508264 | China | USDA | HGL16 | TauL1b |
| 125 | *Aegilops tauschii* Coss. | IG 47259 | Syria | ICARDA | HGL7 | TauL1x |
| 126 | *Aegilops tauschii* Coss. | IG 48042 | India | ICARDA | HGL7 | TauL1x |
| 127 | *Aegilops tauschii* Coss. | IG 48508 | Turkmenistan | ICARDA | HGL7 | TauL1x |
| 128 | *Aegilops tauschii* Coss. | KU-2122 | Iran | KYOTO/NBRP | HGL7 | TauL1x |
| 129 | *Aegilops tauschii* Coss. | KU-2153 | Iran | KYOTO/NBRP | HGL7 | TauL1x |
| 130 | *Aegilops tauschii* Coss. | KU-2157 | Iran | KYOTO/NBRP | HGL7 | TauL1x |
| 131 | *Aegilops tauschii* Coss. | KU-2068 | Iran | KYOTO/NBRP | HGL16 | TauL1x |
| 132 | *Aegilops tauschii* Coss. | KU-2154 | Iran | KYOTO/NBRP | HGL16 | TauL1x |
| 133 | *Aegilops tauschii* Coss. | KU-2619 | Afghanistan | KYOTO/NBRP | HGL16 | TauL1x |
| 134 | *Aegilops tauschii* Coss. | AE 1037 | Georgia | IPK | HGL7 | TauL2a |
| 135 | *Aegilops tauschii* Coss. | IG 120863 | Dagestan | ICARDA | HGL7 | TauL2a |
| 136 | *Aegilops tauschii* Coss. | IG 47173 | Armenia | ICARDA | HGL7 | TauL2a |
| 137 | *Aegilops tauschii* Coss. | IG 47199 | Azerbaijan | ICARDA | HGL7 | TauL2a |
| 138 | *Aegilops tauschii* Coss. | IG 47204 | Azerbaijan | ICARDA | HGL7 | TauL2a |
| 139 | *Aegilops tauschii* Coss. | IG 48274 | Dagestan | ICARDA | HGL7 | TauL2a |
| 140 | *Aegilops tauschii* Coss. | KU-20-1 | Dagestan | KYOTO/NBRP | HGL7 | TauL2a |
| 141 | *Aegilops tauschii* Coss. | KU-20-7 | Iran | KYOTO/NBRP | HGL7 | TauL2a |
| 142 | *Aegilops tauschii* Coss. | KU-20-8 | Iran | KYOTO/NBRP | HGL7 | TauL2a |
| 143 | *Aegilops tauschii* Coss. | KU-2083 | Iran | KYOTO/NBRP | HGL7 | TauL2a |
| 144 | *Aegilops tauschii* Coss. | KU-2086 | Iran | KYOTO/NBRP | HGL7 | TauL2a |
| 145 | *Aegilops tauschii* Coss. | KU-2111 | Iran | KYOTO/NBRP | HGL7 | TauL2a |
| 146 | *Aegilops tauschii* Coss. | KU-2112 | Iran | KYOTO/NBRP | HGL7 | TauL2a |
| 147 | *Aegilops tauschii* Coss. | KU-2118 | Iran | KYOTO/NBRP | HGL7 | TauL2a |
| 148 | *Aegilops tauschii* Coss. | KU-2124 | Iran | KYOTO/NBRP | HGL7 | TauL2a |
| 149 | *Aegilops tauschii* Coss. | KU-2126 | Iran | KYOTO/NBRP | HGL7 | TauL2a |
| 150 | *Aegilops tauschii* Coss. | KU-2155 | Iran | KYOTO/NBRP | HGL7 | TauL2a |
| 151 | *Aegilops tauschii* Coss. | KU-2156 | Iran | KYOTO/NBRP | HGL7 | TauL2a |
| 152 | *Aegilops tauschii* Coss. | KU-2804 | Azerbaijan | KYOTO/NBRP | HGL7 | TauL2a |
| 153 | *Aegilops tauschii* Coss. | KU-2811 | Armenia | KYOTO/NBRP | HGL7 | TauL2a |
| 154 | *Aegilops tauschii* Coss. | PI 486267 | Turkey | USDA | HGL7 | TauL2a |
| 155 | *Aegilops tauschii* Coss. | IG 126991 | Armenia | ICARDA | HGL9 | TauL2a |
| 156 | *Aegilops tauschii* Coss. | IG 47193 | Azerbaijan | ICARDA | HGL9 | TauL2a |
| 157 | *Aegilops tauschii* Coss. | KU-2806 | Azerbaijan | KYOTO/NBRP | HGL9 | TauL2a |
| 158 | *Aegilops tauschii* Coss. | KU-2827 | Georgia | KYOTO/NBRP | HGL9 | TauL2a |
| 159 | *Aegilops tauschii* Coss. | KU-2835B | Georgia | KYOTO/NBRP | HGL9 | TauL2a |
| 160 | *Aegilops tauschii* Coss. | IG 47202 | Azerbaijan | ICARDA | HGL7 | TauL2b |
| 161 | *Aegilops tauschii* Coss. | KU-2069 | Iran | KYOTO/NBRP | HGL7 | TauL2b |
| 162 | *Aegilops tauschii* Coss. | KU-2093 | Iran | KYOTO/NBRP | HGL7 | TauL2b |
| 163 | *Aegilops tauschii* Coss. | KU-2097 | Iran | KYOTO/NBRP | HGL7 | TauL2b |
| 164 | *Aegilops tauschii* Coss. | KU-2104 | Iran | KYOTO/NBRP | HGL7 | TauL2b |
| 165 | *Aegilops tauschii* Coss. | KU-2109 | Iran | KYOTO/NBRP | HGL7 | TauL2b |
| 166 | *Aegilops tauschii* Coss. | KU-2158 | Iran | KYOTO/NBRP | HGL7 | TauL2b |
| 167 | *Aegilops tauschii* Coss. | KU-2159 | Iran | KYOTO/NBRP | HGL7 | TauL2b |
| 168 | *Aegilops tauschii* Coss. | IG 46623 | Syria | ICARDA | HGL9 | TauL2b |
| 169 | *Aegilops tauschii* Coss. | KU-20-10 | Iran | KYOTO/NBRP | HGL9 | TauL2b |
| 170 | *Aegilops tauschii* Coss. | KU-2088 | Iran | KYOTO/NBRP | HGL9 | TauL2b |
| 171 | *Aegilops tauschii* Coss. | KU-2090 | Iran | KYOTO/NBRP | HGL9 | TauL2b |
| 172 | *Aegilops tauschii* Coss. | KU-2091 | Iran | KYOTO/NBRP | HGL9 | TauL2b |
| 173 | *Aegilops tauschii* Coss. | KU-2092 | Iran | KYOTO/NBRP | HGL9 | TauL2b |
| 174 | *Aegilops tauschii* Coss. | KU-2096 | Iran | KYOTO/NBRP | HGL9 | TauL2b |
| 175 | *Aegilops tauschii* Coss. | KU-2098 | Iran | KYOTO/NBRP | HGL9 | TauL2b |
| 176 | *Aegilops tauschii* Coss. | KU-2100 | Iran | KYOTO/NBRP | HGL9 | TauL2b |
| 177 | *Aegilops tauschii* Coss. | KU-2101 | Iran | KYOTO/NBRP | HGL9 | TauL2b |
| 178 | *Aegilops tauschii* Coss. | KU-2102 | Iran | KYOTO/NBRP | HGL9 | TauL2b |
| 179 | *Aegilops tauschii* Coss. | KU-2103 | Iran | KYOTO/NBRP | HGL9 | TauL2b |
| 180 | *Aegilops tauschii* Coss. | KU-2105 | Iran | KYOTO/NBRP | HGL9 | TauL2b |
| 181 | *Aegilops tauschii* Coss. | KU-2106 | Iran | KYOTO/NBRP | HGL9 | TauL2b |
| 182 | *Aegilops tauschii* Coss. | KU-2107 | Iran | KYOTO/NBRP | HGL9 | TauL2b |
| 183 | *Aegilops tauschii* Coss. | KU-2108 | Iran | KYOTO/NBRP | HGL9 | TauL2b |
| 184 | *Aegilops tauschii* Coss. | KU-2160 | Iran | KYOTO/NBRP | HGL9 | TauL2b |
| 185 | *Aegilops tauschii* Coss. | IG 47182 | Azerbaijan | ICARDA | HGL7 | TauL2x |
| 186 | *Aegilops tauschii* Coss. | IG 47186 | Azerbaijan | ICARDA | HGL7 | TauL2x |
| 187 | *Aegilops tauschii* Coss. | IG 47188 | Azerbaijan | ICARDA | HGL7 | TauL2x |
| 188 | *Aegilops tauschii* Coss. | IG 47192 | Azerbaijan | ICARDA | HGL7 | TauL2x |
| 189 | *Aegilops tauschii* Coss. | IG 47194 | Azerbaijan | ICARDA | HGL7 | TauL2x |
| 190 | *Aegilops tauschii* Coss. | IG 47203 | Azerbaijan | ICARDA | HGL7 | TauL2x |
| 191 | *Aegilops tauschii* Coss. | KU-2074 | Iran | KYOTO/NBRP | HGL7 | TauL2x |
| 192 | *Aegilops tauschii* Coss. | KU-2075 | Iran | KYOTO/NBRP | HGL7 | TauL2x |
| 193 | *Aegilops tauschii* Coss. | KU-2077 | Iran | KYOTO/NBRP | HGL7 | TauL2x |
| 194 | *Aegilops tauschii* Coss. | KU-2110 | Iran | KYOTO/NBRP | HGL7 | TauL2x |
| 195 | *Aegilops tauschii* Coss. | KU-2801 | Azerbaijan | KYOTO/NBRP | HGL7 | TauL2x |
| 196 | *Aegilops tauschii* Coss. | IG 120866 | Dagestan | ICARDA | HGL9 | TauL2x |
| 197 | *Aegilops tauschii* Coss. | KU-20-9 | Iran | KYOTO/NBRP | HGL9 | TauL2x |
| 198 | *Aegilops tauschii* Coss. | KU-2076 | Iran | KYOTO/NBRP | HGL9 | TauL2x |
| 199 | *Aegilops tauschii* Coss. | KU-2078 | Iran | KYOTO/NBRP | HGL9 | TauL2x |
| 200 | *Aegilops tauschii* Coss. | KU-2079 | Iran | KYOTO/NBRP | HGL9 | TauL2x |
| 201 | *Aegilops tauschii* Coss. | KU-2080 | Iran | KYOTO/NBRP | HGL9 | TauL2x |
| 202 | *Aegilops tauschii* Coss. | AE 454 | Georgia | IPK | HGL17 | TauL3 |
| 203 | *Aegilops tauschii* Coss. | AE 457 | Georgia | IPK | HGL17 | TauL3 |
| 204 | *Aegilops tauschii* Coss. | AE 929 | Georgia | IPK | HGL17 | TauL3 |
| 205 | *Aegilops tauschii* Coss. | KU-2829A | Georgia | KYOTO/NBRP | HGL17 | TauL3 |
| 206 | *Aegilops tauschii* Coss. | KU-2832 | Georgia | KYOTO/NBRP | HGL17 | TauL3 |
