# Supplementary figures and images for: Intraspecific lineage divergence and its association with reproductive trait change during species range expansion in central Eurasian wild wheat Aegilops tauschii Coss. (Poaceae)
Source: BMC Evol Biol. 2015 Sep 30;15:213. doi: 10.1186/s12862-015-0496-9 (PMC4589133; doi:10.1186/s12862-015-0496-9)

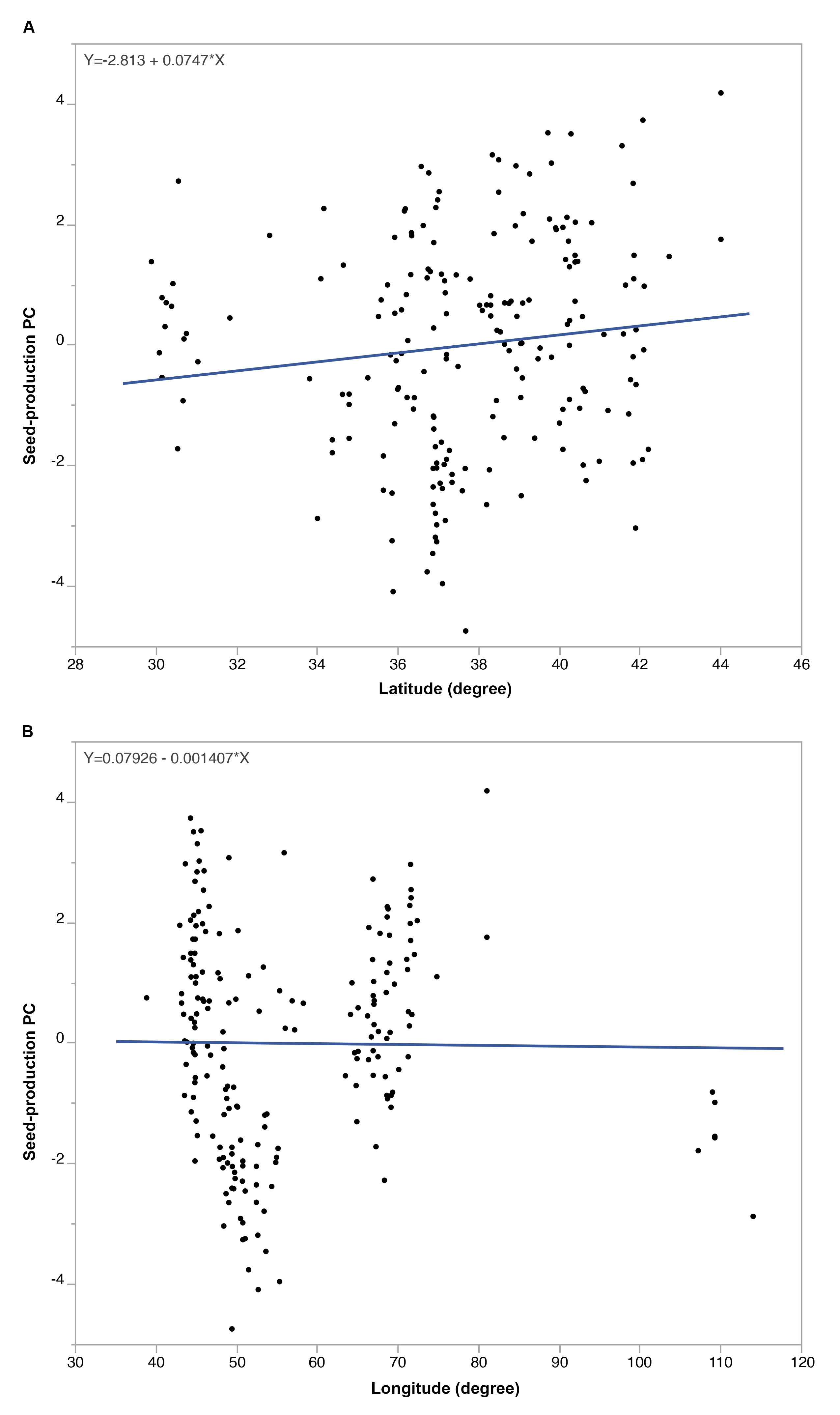

Supplement: Additional file 2: Figure S1. — Plot of the 198 Ae. tauschii accession geographic coordinates against the seed-production PC values. A. Latitude. B. Longitude. (TIFF 32105 kb) [file 12862_2015_496_MOESM2_ESM.tif]
